# In response to: Treatment of patients with multiple myeloma progressing on frontline therapy with lenalidomide, Moreau et al., 2019

**DOI:** 10.1038/s41408-019-0250-4

**Published:** 2019-11-19

**Authors:** Zandra Klippel, Amy Kimball, Christina Tekle

**Affiliations:** Amgen, Inc., Thousand Oaks, CA USA

**Keywords:** Drug development, Myeloma

Dear Editor,

The question of response to multiple myeloma therapy after progression on frontline lenalidomide combination therapy is important. This topic was recently reviewed by Moreau et al. in “Treatment of patients with multiple myeloma progressing on frontline therapy with lenalidomide”^1^. We suggest the following changes be made to align with published literature. When referring to the ENDEAVOR trial comparing carfilzomib vs. bortezomib (both in combination with dexamethasone; Kd vs. Vd), the authors reported the number of patients who were refractory to lenalidomide as 51 in the Kd arm and 45 in the Vd arm. The correct number of lenalidomide-refractory patients is 113 for the Kd arm and 122 for Vd arm^2,3^. When referring to the EMN011 study, the authors reported the dose of carfilzomib for the KPd regimen as 56 mg/m^2^. The correct dose for carfilzomib in the EMN011 study is 20/36 mg/m^2^ ^4^.

We appreciate the author’s presentation and review of the available data on outcomes for lenalidomide-refractory patients and discussion of the limitations of the data. OS data for lenalidomide-refractory patients in ENDEAVOR have been published as an abstract and manuscript^5,6^. There was a 7.8-mo improvement in OS when lenalidomide-refractory patients in ENDEAVOR were treated with Kd vs. Vd (median OS, 29.2 mo vs. 21.4 mo; HR = 0.857; 95% CI: 0.623–1.178)^5,6^. The ENDEAVOR study enrolled nearly as many lenalidomide-refractory patients (*n* = 233) as the phase 3 OPTIMISMM study (*n* = 238), adding to the available data on approved regimens for patients progressing on lenalidomide treatment.
